# Live-Attenuated Vaccines in Pediatric Solid Organ Transplant

**DOI:** 10.3390/vaccines12040376

**Published:** 2024-04-01

**Authors:** Christopher Hartley, Tina Thomas, Sara Kathryn Smith, Wikrom Karnsakul

**Affiliations:** 1 The Department of Pharmacy, The Johns Hopkins Hospital, Baltimore, MD 21287, USA; 2 Pediatric Liver Center, The Department of Pediatrics, The Johns Hopkins Hospital, Baltimore, MD 21287, USA

**Keywords:** live-attenuated vaccines, prophylaxis, measles, mumps, rubella, varicella, pediatric solid organ transplant

## Abstract

Measles, mumps, rubella (MMR), and varicella incidence rates have increased due to the delayed vaccination schedules of children secondary to the COVID-19 pandemic. Decreased herd immunity creates a risk for immunocompetent children and immunocompromised individuals in the community. Historically, live-attenuated vaccines (MMR and varicella) were recommended before solid organ transplants. The amount of time before transplant when this is appropriate is often debated, as is the utility of vaccine titers. MMR and varicella vaccines previously were not recommended in immunocompromised patients post-solid organ transplant due to the undue risk of transmission and posed infection risk. The new literature on live-attenuated vaccines in post-transplant pediatric patients provides more insight into the vaccines’ safety and efficacy. The present article aims to provide guidance on live-attenuated vaccines (MMR and varicella) in the pre-transplant and post-operative solid organ transplant phases of care in pediatric patients.

## 1. Introduction

Immunosuppression is necessary for solid organ transplant (SOT) recipients; however, many recipients are not completely vaccinated with routine childhood vaccinations for various reasons (age at time of transplant, access to primary care providers, distrust in the medical system, etc.). A risk of immunosuppression is that previously vaccinated patients often lose seroprotection, which is necessary to defend against vaccine-preventable diseases. The need for herd immunity (defined for measles as >95% of the population to prevent sustained transmission) [1] is imperative to avoid serious sequelae in immunocompromised patients. Herd immunity has declined in recent years secondary to anti-vaccination movements and children being behind on routine immunization schedules due to the COVID-19 pandemic. In 2022, 83% of the world’s population had only received their first measles vaccination by their first birthday, which was the lowest population percentage of measles vaccination since 2008 [2]. Although underreported, outbreaks of select infectious diseases (measles, varicella, etc.) are reportable events to local and state public health departments. The *Journal of Infectious Diseases* reviewed reported varicella outbreaks from 2016 to 2019, which affected 5135 patients in the United States. Of these patients, 79% were not appropriately vaccinated (either unvaccinated or under-vaccinated for age) [3]. Between 1 January and 1 October 2019, 1429 patients were affected in 22 measles outbreaks in the United States. A risk factor for vaccine-preventable illnesses (VPI) outbreaks includes unvaccinated/under-vaccinated individuals living in close-knit communities [4,5]. Outbreaks afflict not only individuals in tight-knit communities but also larger populations (where individuals may not be known to be immunosuppressed by those carrying the infectious disease).

Historically, live-attenuated vaccines (measles, mumps, rubella, and varicella) have not been recommended after SOT due to the perceived risk of disease development. However, transplant recipients are at a higher risk for fatal complications from measles, mumps, and varicella. Severe varicella infections in transplant recipients, despite optimal treatment, are of significant concern, and vaccinating these patients could prevent morbidity and mortality [6,7]. Immunosuppressed individuals lack typical signs and symptoms of the disease compared to others who are immunocompetent, leading to delayed diagnosis and treatment [6,7]. Measles generally presents with cough, coryza, conjunctivitis, and maculopapular rash; however, about 25–50% of immunosuppressed patients present without a rash, and many develop severe complications of pneumonitis or encephalitis. The serious complication rate in immunocompromised patients is approximately 80%; of these, 40% are fatal [8]. Patients following SOT are at risk of systemic illness with high morbidity and mortality rates, acute and cellular graft rejection, and graft injury [9].

The incidence of hospitalization from vaccine-preventable infections in children following SOT was 87 times higher than that in the general population, with hospitalization occurring in 15% of SOT recipients within five years post-operatively [9]. New literature has emerged on utilizing live-attenuated vaccines after SOT. This paper aims to discuss vaccinating patients to prevent vaccine-preventable illnesses in SOT recipients.

## 2. Overview of Live Vaccines

The measles, mumps, and rubella (MMR) vaccine was first approved by the United States Food and Drug Administration (FDA) in 1971 and is under the trade names MMR-II^®^ and Priorix^®^ [10]. The varicella vaccine was FDA-approved in 1995 and is under the trade name Varivax^®^ [11]. Both vaccines are part of a two-dose series. The United States Department of Health and Human Services (HHS) and Centers for Disease Control and Prevention (CDC) immunization recommendations from the Advisory Committee on Immunization Practices (ACIP) list the minimum age for the MMR and varicella vaccines as 12 months. Ideally, both vaccines should be given at 12–15 months of age for the first dose and then 4–6 years for the second dose. Of note, the incubation period (time from exposure to onset of symptoms) for measles is 21 days; for mumps, 28 days; for rubella, 14 days; and for varicella, three months [12]. The minimum duration between doses per the ACIP is four weeks for MMR and four weeks for varicella [13]. The ACIP recommends against MMR and varicella vaccines for immunocompromised status patients (excluding Human Immunodeficiency Virus), stating that it is contraindicated to vaccinate these individuals [13]. The Infectious Diseases Society of America (IDSA) and the American Society of Transplantation Infectious Diseases Community of Practice (AST IDCOP) have also released this guidance [6,14,15,16,17,18,19].

Feldman and colleagues conducted an email survey regarding vaccinations by transplant hepatologists in the United States and Canada participating in The North American Society for Pediatric Gastroenterology, Hepatology, and Nutrition (SPLIT). In their survey, they found that in the pre-transplant phase, live vaccines were recommended always by 15% of hepatologists, sometimes by 84%, and never by 1%. They also found that only 6% of pre-transplant clinics administered all needed vaccines, 53% administered some, and 41% administered no vaccines [9]. Administering vaccines within a transplant clinic would significantly improve immunization compliance.

Rotavirus is part of patients’ routine immunization schedule as a two-dose series to be given four weeks apart, as early as two months of age, but it is not recommended after age 24 weeks. Therefore, it would not be clinically indicated in most SOT cases given the age of recipients. Other special-situation liver vaccinations include yellow fever, oral typhoid, Bacille Calmette–Guerin (BCG), and smallpox. These special-situation vaccines should be considered for transplant candidates based on travel and whether the patient is in an area endemic for such diseases. The CDC provides guidelines on when each vaccine is indicated for immunocompetent persons [13]. An important future area of research would involve reviewing case reports relating to other live vaccines (specifically, the special-situations vaccines) in immunocompromised patients.

## 3. Pre-Transplant Vaccination

Both the IDSA and AST IDCOP recommend utilizing all age-, medical-, and exposure-appropriate vaccinations following the United States HHS and CDC immunization recommendations from the Advisory Committee on Immunization Practices (ACIP). These bodies recommend vaccination before SOT [13,20,21]. The AST IDCOP recommends assessing the vaccination status of all patients at their first transplant clinic visits and again when patients are actively listed for transplant at an institution [20,21]. Close contacts of transplant recipients are recommended to receive MMR and varicella vaccines to provide household immunity with a low transmission risk from the live-attenuated vaccine to the transplant recipient [21]. The listing laboratory parameters required for the SOT active listing include a tuberculosis skin test (TST). Administering and interpreting a TST test within four weeks of live-attenuated vaccines can produce false negative results [22]. Therefore, it is imperative that the patients have the TST completed the same day that they receive the MMR or varicella vaccines or wait for four more weeks after live virus vaccination.

The minimum age for the first dose of the MMR vaccine is six months [20,21]. The AST IDCOP recommends that the varicella vaccine be given as early as nine months. However, per the IDSA, it may be given as early as six months of age. The time between doses for varicella is ideally at least three months apart; however, to get patients seropositive or fully vaccinated before transplant, they may receive the second dose of varicella vaccine within four weeks of the last dose as long as they are still not anticipated to receive a vaccine within four weeks of transplant [21]. If these live-attenuated vaccines are given before one year of age and patients are not transplanted or anticipated to be transplanted within the next four weeks, they should receive a repeat series of MMR and varicella. This repetition is because maternal antibodies interfere with the response to live vaccines [21]. The one-year-of-age recommendation is because maternal antibodies have waned by then. One year also aligns with the twelve-month recommendation from ACIP for routinely scheduled vaccinations in pediatric patients [13,21].

Live-attenuated vaccines are recommended at least four weeks prior to transplant to allow for the resolution of vaccine-related viral replication [21]. Viral replication and the development of vaccines generally is less than three weeks, which is why the 2013 IDSA recommendation states that live-attenuated vaccines should be given at least four weeks before the anticipated transplant. IDSA guidance states that the primary vaccination could take longer to have a robust immune response [20]. A case report by Rosenthal and colleagues evaluated five SOT patients who received either MMR or Varicella vaccine eight to 21 days before liver (2) and heart (3) transplants. All patients received methylprednisolone during induction. Four patients received anti-thymocyte globulin during induction. One patient received both anti-thymocyte globulin and basiliximab. One patient who did not receive anti-thymocyte globulin received just methylprednisolone and tacrolimus. Four patients received immunoglobulin (IVIG) for prophylaxis; two received IVIG plus antiviral therapy (ganciclovir or acyclovir). No patients in this report developed vaccine-related viral illness after transplant [23].

## 4. Post-Transplant Vaccination

Several case reports and cohort studies have discussed post-transplant vaccination in pediatric patients. Differences in induction immunosuppressive therapy can vary drastically based on the organ(s) being transplanted, panel-reactive antibody (PRA), the institution, and the attending provider/surgeon. Generally, patients receive, at minimum, high-dose corticosteroids, an antiproliferative agent, and calcineurin inhibitors. In addition, patients can receive different T cell-depleting agents (thymoglobulin, basiliximab, etc.). Seroprotection can be assessed using antibody titers, which decrease over time, especially when a patient is on immunosuppression. An article by Kano and colleagues discussed post-operative antibody titers for live-attenuated vaccines in pediatric SOT. Antibodies waned from six months post-transplant to one year post-transplant (measles, mumps, rubella, and varicella 78%, 100%, 81%, and 93% vs. 45%, 94%, 57%, and 75%) [24].

The 2013 IDSA guideline recommends that MMR and varicella vaccines should generally not be administered post-transplant. The following recommendation considers administering the varicella vaccine if the patient is on minimal immunosuppression at the time of evaluation and has no recent graft rejection [20]. The AST-IDCOP recommendation from 2019 states that for certain at-risk populations, MMR and varicella vaccines could be safe and effective [21].

However, these recommendations are based on several smaller studies. Danerseau and colleagues published a review article on the efficacy and safety of live vaccines in patients with immunosuppression. With 206 combined doses of either MMR or varicella vaccines, some patients had post-immunization titers in the immune range after either one (109/171) or two doses (15/22). Of these patients, 112 children were SOT recipients, and most were on tacrolimus, cyclosporine, or prednisone. Some patients were also on mycophenolate or azathioprine. Patients were immunized in a median of one to two years post-transplant [6]. Regarding typical maintenance immunosuppression, it is logical that patients further out from transplant would be on fewer immunosuppressants.

Posfay–Barbe and colleagues studied 77 pediatric liver transplant recipients, most of whom were on either tacrolimus or cyclosporine. A few patients were also on mycophenolate or steroids. Most of the patients (47%) were within two years of transplant, then greater than five years (31%), then in the two-to-five-year range (22%). No patients had breakthrough varicella disease after a median of 4.14 years with no graft rejection episodes. Based on varicella titers at follow-up visits, 22% of patients required a third dose to be seropositive for varicella antibodies [18].

Pittet and colleagues had 90 patients who received a liver transplant and were at least one year out from the transplant. Patients had a median age of 10.3 years (IQR 5.7–13.6 years) at the time of inclusion and a median age of 1.4 years (IQR 0.8–4.1 years) at the time of transplant. The patients were divided into those seroprotected against measles (44/90) versus those not seroprotected against measles (46/90). Of the 46 patients not seroprotected, 39% had been immunized before SOT. Those not seroprotected were given one dose of MMR vaccine, and antibody titers were checked after four weeks. If their antibody titers were low, they received a second dose of MMR vaccine [19].

Of their 90 patients, Pittet and colleagues immunized 40 pediatric liver transplant recipients who were more than one year post-transplant (median 6.3 years, IQR 4–10.9) on low immunosuppression, which is defined in this study as steroids < 2 mg/kg/day, tacrolimus < 0.3 mg/kg/day, and tacrolimus levels < 8 ng/mL for greater than 1 month. They received one dose of the MMR vaccine, and antibody titers were checked after four weeks [19]. Seroprotection rates were 88% and 96% after the first and second MMR vaccines. Of the 40 total patients, 35 of the immunized patients had two-year follow-up results. Almost all the patients (30/35) remained seroprotected; four of the five unprotected patients required a third MMR vaccine dose, and one had already received a third dose. At the three-year follow-up, 16/18 participants were still seroprotected, and the two who lost protection responded well to a booster [19]. Serious adverse effects following MMR immunization were rare. One patient developed alloimmune hepatitis 4.5 months after the vaccine. Three patients had rejection episodes successfully treated at 6 months, 9 months, and 3 years post-immunization. One dose of MMR vaccine yielded seropositive immunity in 89% of patients after four weeks; however, 38% of patients lost their seropositive immunity one year following immunization, requiring additional boosters. No patients in this study developed MMR or died [19].

Weinberg and colleagues reported 19 patients who received the varicella vaccine over six months after a liver transplant or 12 months after a small bowel transplant. None had experienced rejection in the past month. All were on alternating days of prednisone (<0.3 mg/kg) and had a trough tacrolimus level of ≤10 ng/mL. No patients had developed liver enzyme elevations. However, four subjects reported varicella skin rashes, and three were treated with acyclovir. These skin lesions healed within 1–7 days with a median of four days after the treatment. Fifteen patients had varicella antibody titers performed at follow-up visits, and 13/15 (87%) seroconverted at 12 weeks post-vaccination [25].

Kano and colleagues studied the efficacy and safety of immunization on 58 liver transplant children (aged 9 months to 17 years, median age 4 years) at least one year post-liver transplant in their stable condition with normal liver function tests, trough blood concentrations of tacrolimus < 5 ng/mL and cyclosporine < 50 ng/mL, and off corticosteroids longer than 6 months. Post-immunization follow-up time was a median of 30 months (range 6–78 months). Immunity was checked at one, two, and three years, and rates of positive immunity remained at 100% for measles and rubella, 90% for mumps, and 67% for varicella after three years post-immunization. In addition, the authors stated that reimmunization had no side effects [24].

Feldman and colleagues from 18 transplant centers across the United States had a cohort study where they provided 281 pediatric kidney and liver transplant recipients with MMR or varicella vaccines who either did not receive primary MMR and varicella vaccine series or who did not have seroprotective antibody titers at the time of enrollment. The LIVE VAC study aimed to see the development of seroprotective antibodies against MMR and varicella following post-transplant liver vaccination. The median age at first post-transplant vaccine was 8.9 years (IQR 4.7–13.8 years), and the median time from transplant to enrollment was 6.3 years (IQR 3.4–11.1 years). The majority of patients were on low or medium-level immunosuppression (low defined as a monotherapy trough with tacrolimus < 5 ng/mL, sirolimus < 5 ng/mL, cyclosporine < 100 ng/mL; medium defined as two or fewer agents or trough concentration tacrolimus plus sirolimus 5–8 ng/mL or steroids < 0.5 mg/kg/dose). Patient antibody levels were obtained at 0, 3, and 12 months following vaccinations. Only 5% of patients received a T-cell-depleting agent during induction therapy, and 4% received blood products in the past year. Over 75% of patients who initially had seroprotective antibodies to the MMR vaccine at one year post-transplant vaccination were still protected, and the majority of these patients needed one or two vaccines (patients initially seroprotected who maintained at one year; measles, 45/49 (92%), mumps 35/42 (83%), rubella 51/54 (94%)). Varicella rates were lower for those who remained protected with seropositive antibodies one year after the end of vaccination (34/44 (77%)). Antibody levels were obtained at 0 to 3 months and one year after vaccinations. For varicella, patients required two vaccinations to convert to seropositive status post-transplant. For MMR, patients needed one to two vaccines to convert to seropositive status post-transplant. Regarding safety, there were no measles nor rubella cases and no episodes of organ rejection one month after vaccination. One patient had concern for mumps due to transient non-tender subauricular lymph node swelling, which spontaneously resolved without clinician intervention. There were 5/217 (2%) of patients who developed clinical varicella ≥ 7 days post-vaccination who had resolving symptoms after one week. Of these five patients, three of them required antiviral therapy. All five children had medium or high immunosuppression regimens at the time of vaccination. No graft injuries or patient deaths were reported. Some patients did not have one-year titers drawn to assess the primary outcome. The primary limitations of this study were that one year post-vaccine data was missing for some patients and that there was no information past one year of vaccine data [26]. Table 1 summarizes studies of post-transplant recipients who received live vaccines.

Close monitoring of SOT patients for live vaccines includes immediate medical attention following the development of a new rash or fever (>38 degrees C) or unexplained illness or symptoms within four weeks of vaccination. Within four weeks following live vaccination, a medical provider should call to inquire about any symptoms the patient may experience, and in the US, utilize the US Vaccine Adverse Event Reporting System (VAERS) to report adverse effects related to vaccines [27].

## 5. Discussion

The COVID-19 pandemic led to decreased herd immunity secondary to anti-vaccinators and children who were behind on routine vaccinations. By having standard routine immunizations available at transplant clinics, providers could help bring patients current on immunizations and provide immunizations that are beneficial before transplant.

It is vital to review if patients are up-to-date on routine immunizations, and what vaccines are needed, at pre-transplant evaluation. At the time of listing, the multidisciplinary team must review immunization records again. When the team is ready to administer MMR and varicella vaccines, they should ideally place the TST simultaneously (otherwise in four weeks).

Measles, mumps, rubella, and varicella immunizations can be administered at six months of age, and ideally, there should be at least four weeks between the second dose in the series. If patients are less than one year of age, they should be re-vaccinated at one year of age unless they are expected to receive a transplant within the next four weeks. This is due to maternal antibodies interfering with the immune response. Programs rarely remove a patient from active status for live-attenuated vaccines. Blood products such as IVIG, platelets, and whole red blood cells should not be a barrier to obtaining live vaccines, despite recommendations that one should wait between three and eleven months after these for vaccines.

If a patient receives the varicella live vaccine and is offered an organ within four weeks of the vaccination, it appears reasonable to dose them with intravenous immunoglobulin and valacyclovir/ganciclovir. After transplant, seroprotection against MMR and varicella will wane over time, putting immunosuppressed patients at risk of serious sequelae from infection, graft dysfunction, or organ rejection.

An important consideration in transplant is initial vaccination post-transplant for those who did not receive live-attenuated vaccines and boosters and revaccination for those who received MMR and varicella before transplant. If the opportunity to vaccinate prior to transplant is missed or seroprotection wanes after transplantation, it will be crucial to consider vaccinating these vulnerable patients against MMR and varicella.

**Figure 1:**
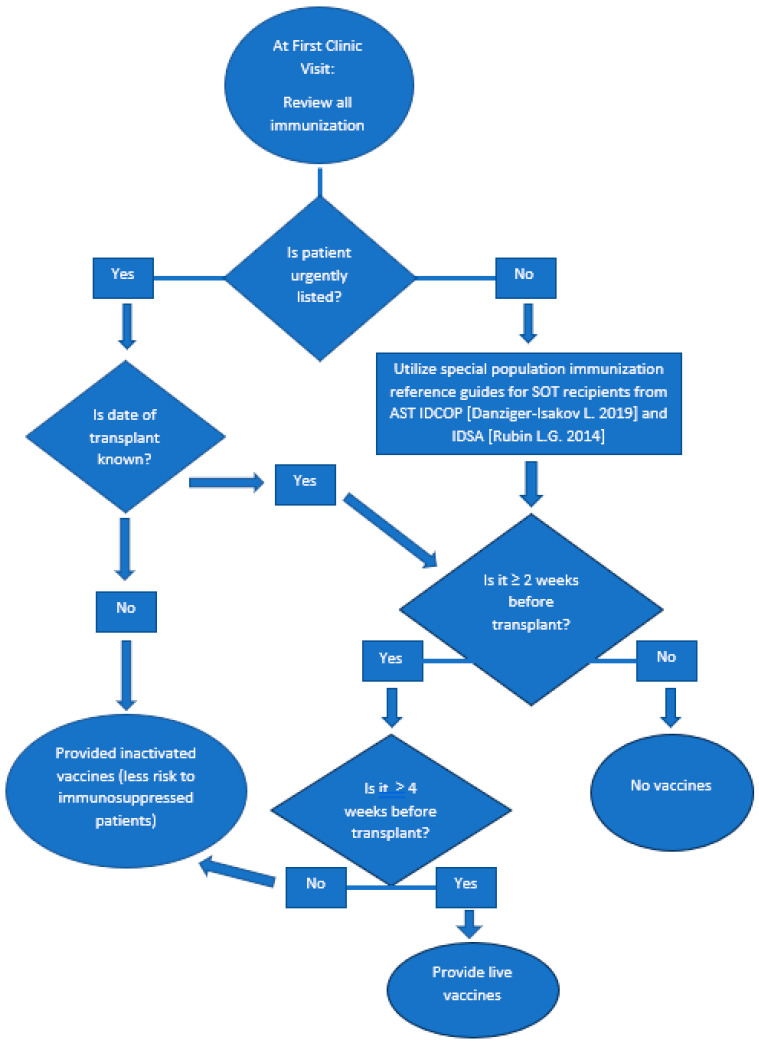
Proposed vaccination plan for patients undergoing SOT. [20,21].

Within appropriate clinical timeframes (see flow chart in Figure 1), changes in cellular and humoral immunity lab values are ways clinicians can guide discussion on live vaccinations after SOT. Parameters include immunoglobulin to prevent hypogammaglobulinemia and whether the patient has antibody-specific levels from a previous vaccination. If the patient has hypogammaglobulinemia, intravenous immunoglobulin could be recommended. If antibody levels are detectable for vaccines, boosters are not recommended. Reviewing lymphocyte subsets such as CD4+ count or percentages could be implemented in SOT recipients for cellular immunity. Patients with HIV are immunosuppressed; however, the CDC ACIP recommends that they receive both MMR and varicella vaccines. CD4+ percentages include ≥ 15% for at least 6 months; if >5 years old, CD4+ percentages ≥ 15% and CD4+ ≥200 lymphocytes/mm^2^ for at least 6 months. For age-specific CD4+ counts, if ≤12 months, they would need a CD4+ count of >750 lymphocytes/mm^3^ and ≥ 500 lymphocytes/mm^3^ if age 1 to 5 [28,29]. Although the use of CD4+ counts is not currently recommended in immunocompromised patients outside of HIV, it would be an interesting area of further research.

## 6. Conclusions

MMR and varicella vaccination in pediatric SOT is a crucial area of transplant care to address, especially with so many patients behind on vaccines secondary to the COVID-19 pandemic and the growing number of anti-vaccinators. The decrease in herd immunity increases the risk for immunocompetent children and immunocompromised patients of all ages. Ideally, MMR and varicella should be administered per ACIP recommendations. Patients requiring transplantation before the first year of life should have TB testing pre-transplantation. They should then receive MMR and varicella immunizations at least at six months of age and a second dose of each four weeks later (if not anticipated to have a transplant within the subsequent four weeks). If the patient is transplanted within the four-week post-vaccination timeframe, it is reasonable to give intravenous immunoglobulin and valacyclovir/ganciclovir to prevent varicella infection.

If patients have not undergone transplantation by the time they reach one year of age, and received early immunizations before that age, they should receive live vaccines if transplantation is not anticipated within the next four weeks.

## Figures and Tables

**Table 1:** Studies of live vaccines in post-transplant recipients.

Study Author	Intervention	Number of Patients	Outcome	Median Time Since Transplant in Years (IQR)	Results
Danerseau Review [6]	MMR and Varicella	112	Safety and Efficacy	Multiple smaller case studies	1 case of rejection 3 weeks post-immunization, 9 possible cases 1 case of varicella, similar to vaccine-associated varicella, which occurs in 10% of normal subjects.
Feldman [26]	MMR and Varicella	281	Safety and Efficacy	8.9 (4.7–13.8)	After final post-transplant vaccines, patients were 92, 83, 94, and 77% seroconverted for measles, mumps, rubella, and varicella No measles or rubella cases within 1 month post-vaccine No organ rejection cases within 1 month post-vaccine One patient with transient non-tender swelling of lymph node, which self-resolved
Kano [24]	MMR and Varicella	58	Safety and Efficacy	Not available; at least one year	Immunity rates were 100, 100, 90, and 67% for measles, mumps, rubella, and varicella three years after immunization No side effects were listed for any patients
Pittet [19]	MMR	40	Safety	6.3 (4–10.9)	No patients developed MMR or died 88% and 96% of patients seroconverted after the first and second vaccination
Posfay-barbe [18]	Varicella	77	Safety and Efficacy	3	5 patients developed vesicles, but these remained isolated and all resolved within 48 h without antiviral therapy 100% of patients seroconverted after three vaccinations
Weinberg [25]	Varicella vaccine in patient at least six months outside of liver transplant or 12 months after small bowel transplant	19	Safety and Efficacy	1.1 (0.7–5.6)	4 patients developed rash, three were treated with acyclovir; all lesions healed within 7 days 13/15 patients seroconverted after 12 weeks post-vaccination
